# *Streptococcus pneumoniae* Transmission Is Blocked by Type-Specific Immunity in an Infant Mouse Model

**DOI:** 10.1128/mBio.00188-17

**Published:** 2017-03-14

**Authors:** Tonia Zangari, Yang Wang, Jeffrey N. Weiser

**Affiliations:** a Department of Microbiology, New York University School of Medicine, New York, New York, USA; b School of Medicine, Tsinghua University, Beijing, China; University of Mississippi Medical Center

**Keywords:** PCV, *Streptococcus pneumoniae*, immunity, transmission

## Abstract

Epidemiological studies on *Streptococcus pneumoniae* show that rates of carriage are highest in early childhood and that the major benefit of the pneumococcal conjugate vaccine (PCV) is a reduction in the incidence of nasopharyngeal colonization through decreased transmission within a population. In this study, we sought to understand how anti-*S. pneumoniae* immunity affects nasal shedding of bacteria, the limiting step in experimental pneumococcal transmission. Using an infant mouse model, we examined the role of immunity (passed from mother to pup) on shedding and within-litter transmission of *S. pneumoniae* by pups infected at 4 days of life. Pups from both previously colonized immune and PCV-vaccinated mothers had higher levels of anti-*S. pneumoniae* IgG than pups from non-immune or non-vaccinated mothers and shed significantly fewer *S. pneumoniae* over the first 5 days of infection. By setting up cross-foster experiments, we demonstrated that maternal passage of antibody to pups either *in utero* or post-natally decreases *S. pneumoniae* shedding. Passive immunization experiments showed that type-specific antibody to capsular polysaccharide is sufficient to decrease shedding and that the agglutinating function of immunoglobulin is required for this effect. Finally, we established that anti-pneumococcal immunity and anti-PCV vaccination block host-to-host transmission of *S. pneumoniae*. Moreover, immunity in either the donor or recipient pups alone was sufficient to reduce rates of transmission, indicating that decreased shedding and protection from acquisition of colonization are both contributing factors. Our findings provide a mechanistic explanation for the reduced levels of *S. pneumoniae* transmission between hosts immune from prior exposure and among vaccinated children.

## IMPORTANCE

Rates of carriage of the bacterial pathogen *Streptococcus pneumoniae* are highest among young children, and this is the target group for the pneumococcal conjugate vaccine (PCV). Epidemiological studies have suggested that a major benefit of the PCV is a reduction in host-to-host transmission, which also protects the non-vaccinated population (“herd immunity”). In this study, we examined the role of anti-pneumococcal immunity on nasal shedding and transmission of the pathogen using an infant mouse model. We found that shedding is decreased and transmission is blocked by anti-pneumococcal immunity and PCV vaccination. Additionally, transmission rates decreased if either the infected or contact pups were immune, indicating that reduced shedding and protection from the establishment of colonization are both contributing factors. Our study provides a mechanistic explanation for the herd immunity effect seen after the introduction of PCV and identifies potential points of intervention, which may have implications for future vaccine development.

## INTRODUCTION

A key step in the life cycle of pathogens is transmission from one host to another. In general, this process involves colonization of host surfaces, exit (“shedding”), and acquisition and establishment of the organism by a new, susceptible host. An example of this paradigm is the Gram-positive bacterial pathogen *Streptococcus pneumoniae*, which colonizes the mucosal surfaces of the human nasopharynx and is shed in nasal and oral secretions. Pneumococcal transmission among the human population occurs through close contact with upper respiratory tract (URT) secretions of colonized individuals (1).

The estimated global burden of serious pneumococcal disease is 14.5 million cases (2), and the WHO estimates that 476,000 deaths of children (<5 years old) occurred in 2008 due to pneumococcal infections (WHO/IVB 2012). Young children are the main reservoir of *S. pneumoniae* (3), although healthy adult carriers also serve as a reservoir and as a source for transmission to other hosts (4).

In 2000, the first pneumococcal conjugate vaccine (PCV) was licensed for use in the United States and included the seven most common capsular polysaccharide serotypes responsible for invasive pneumococcal disease in children. Extended versions now include up to 13 of the more than 90 known serotypes (5). These vaccines are approved for use in children as young as 6 weeks old to protect against pneumococcal disease in early infancy (6). Numerous epidemiological studies on the impact of PCVs have indicated that their major contribution to public health is due to the indirect effect of vaccination (herd immunity), by which reduced carriage of *S. pneumoniae* in vaccinated children decreases pneumococcal transmission to vulnerable unvaccinated groups, especially the elderly (7–10). However, protection by PCV is incomplete as most pneumococcal serotypes are not included in the current vaccine formulations.

Although pneumococcal disease and colonization have been extensively studied in animal models, there is still little known about transmission of this common pathogen. In this study, we sought to understand how anti-*S. pneumoniae* immunity affects transmission between hosts. Our prior studies have shown that a critical factor in transmission is the number of pneumococci shed from the nasopharynx of the infected host: those strains that shed at high levels also have higher intralitter transmission rates in an infant mouse model (11, 12). Factors that increase *S. pneumoniae* shedding, such as high-density colonization, influenza A virus (IAV) coinfection (11), and Toll-like receptor 2 (TLR2) deficiency in the context of IAV coinfection (13), also increase transmission rates. High shedding is required for the organism to prevail over a tight population bottleneck that exists in transmission between hosts (13). Similarly, pneumococcal strains that shed at high rates in the absence of IAV transmit at detectable levels in an infant mouse model (12). However, in this model, no host factors have yet been identified that decrease shedding and transmission without impacting colonization density. With the concept of herd immunity in mind, we hypothesize that anti-pneumococcal immunity, induced by prior colonization or vaccination, would decrease the number of pneumococci shed from an infected pup and thereby decrease transmission of the pathogen to littermates.

## RESULTS

### *S. pneumoniae* infection induced antibody that reduces bacterial shedding.

We utilized an infant mouse model (11, 12) to assess the effect of anti-pneumococcal immunity on colonization and shedding of *S. pneumoniae*. The pups were infected on day 4 of life, as we previously established that pups infected on later days do not shed *S. pneumoniae* as highly as those infected on day 4. However, as there was not sufficient time to develop an adaptive immune response before infection in 4-day-old pups, it was necessary to induce an anti-pneumococcal immune response in adult female mice that would pass antibodies to their pups. Female mice were immunized by intranasal (i.n.) infection with an *S. pneumoniae* isolate of serotype 4 (T4) or 23F (T23F). At least 4 weeks post-infection (p.i.), their litters were utilized in shedding and colonization studies. At the time that the pups are infected, the dams were no longer colonized, as previous data show that adult mice fully clear nasopharyngeal colonization of *S. pneumoniae* by 21 days p.i. (14). Furthermore, preliminary assessment indicated that pups were not infected from their mothers before receiving the inoculum on day 4 of life.

**FIG 1:**
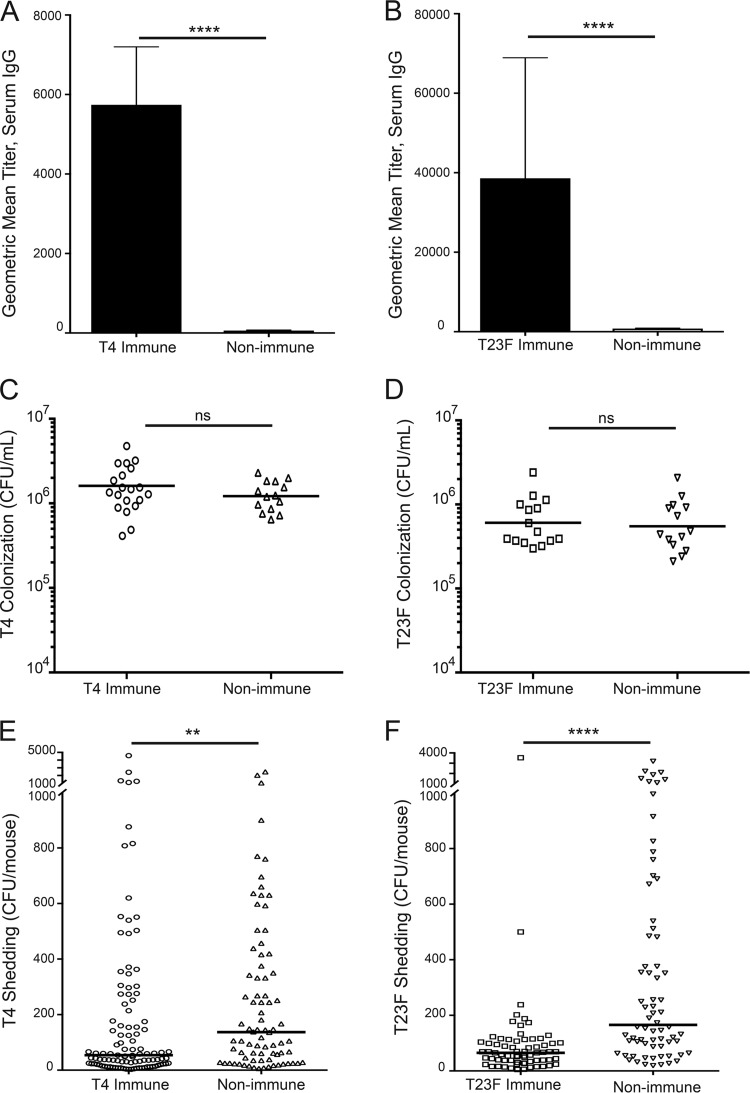
*S. pneumoniae* infection induces an anti-pneumococcal IgG serum response in dams, transferred to their pups, which reduces bacterial shedding but does not impact colonization. (A) Pups from T4-immunized mothers had a higher anti-*S. pneumoniae* serum IgG titer, as assessed by ELISA, than pups from non-immune mothers. (B) Similarly, pups from T23F-infected mothers had higher anti-T23F *S. pneumoniae* serum IgG titers than pups from non-immune mothers. All pups were infected on day 4 of life, and serum was collected 5 days p.i. All ELISA data are represented as mean values with standard errors of the means (SEM). Pups were infected on day 4 of life, and colonization was assessed by culture of URT lavages on day 5 p.i. *S. pneumoniae* T4 (C) and T23F (D) colonization of pups from immune mothers did not differ from *S. pneumoniae* colonization of pups from non-immune mothers. Pneumococcal shedding samples, measured by culturing nasal secretions, were collected on days 1 to 5 p.i., and results are shown as median shedding, with each symbol representing the value from an individual pup on a single day. Pups from immune mothers shed significantly less T4 (E) and T23F (F) than pups from non-immune mothers. *n* ≥ 14 pups/group. ns, not significant; **, *P* < 0.01; ****, *P* < 0.0001 (Mann-Whitney test).

Pups were infected on day 4 of life with T4 or T23F, and sera were collected 5 days p.i., to assess anti-pneumococcal IgG by an enzyme-linked immunosorbent assay (ELISA) using whole bacteria (in which the plates were coated with the same strain). Prior maternal infection induced an anti-pneumococcal response in mothers that is passed to their litters, as immune pups had significantly higher anti-T4 (Fig. 1A) and anti-T23F (Fig. 1B) serum IgG titers than non-immune pups. Levels of anti-*S. pneumoniae* IgG were similar in dams and their pups. We were unable to detect an anti-*S. pneumoniae* serum IgA titer in these pups. Colonization was measured on day 5 p.i.; samples were collected by upper respiratory tract (URT) lavage and plated to determine colonization density. Anti-*S. pneumoniae* titers did not impact pneumococcal colonization, as T4 and T23F colonization of pups from immune mothers did not differ from *S. pneumoniae* colonization of pups from unimmunized mothers (Fig. 1C and D). Although the airway surface fluid is heavily diluted while obtaining URT lavage samples, anti-*S. pneumoniae* IgG could be detected in nasal lavage samples from immune mice by whole-cell ELISA. *S. pneumoniae* shedding from the nares of pups was enumerated on days 1 to 5 p.i. Despite similar colonization levels, pups from immunized mothers showed significantly lower T4 (Fig. 1E) and T23F (Fig. 1F) shedding compared to pups from nonimmunized mothers, indicating a role for anti-pneumococcal antibody in decreased *S. pneumoniae* shedding.

### PCV13 induced antibody that reduces bacterial shedding.

As pneumococcal infection induces an immune response sufficient to decrease *S. pneumoniae* shedding upon subsequent exposure to the same serotype, we next assessed the impact of the pneumococcal conjugate vaccine PCV13 (Prevnar13; Pfizer) on *S. pneumoniae* shedding and colonization. Throughout this study, the term “vaccinated” will refer to mothers that were treated with PCV13 and their pups, whereas “immunized” or “immune” refers to mothers that were infected with *S. pneumoniae* prior to breeding and their litters. Serum IgG titers, colonization, and shedding were assessed in pups from vaccinated (PCV13) mothers and compared to those of pups from non-vaccinated mothers.

**FIG 2:**
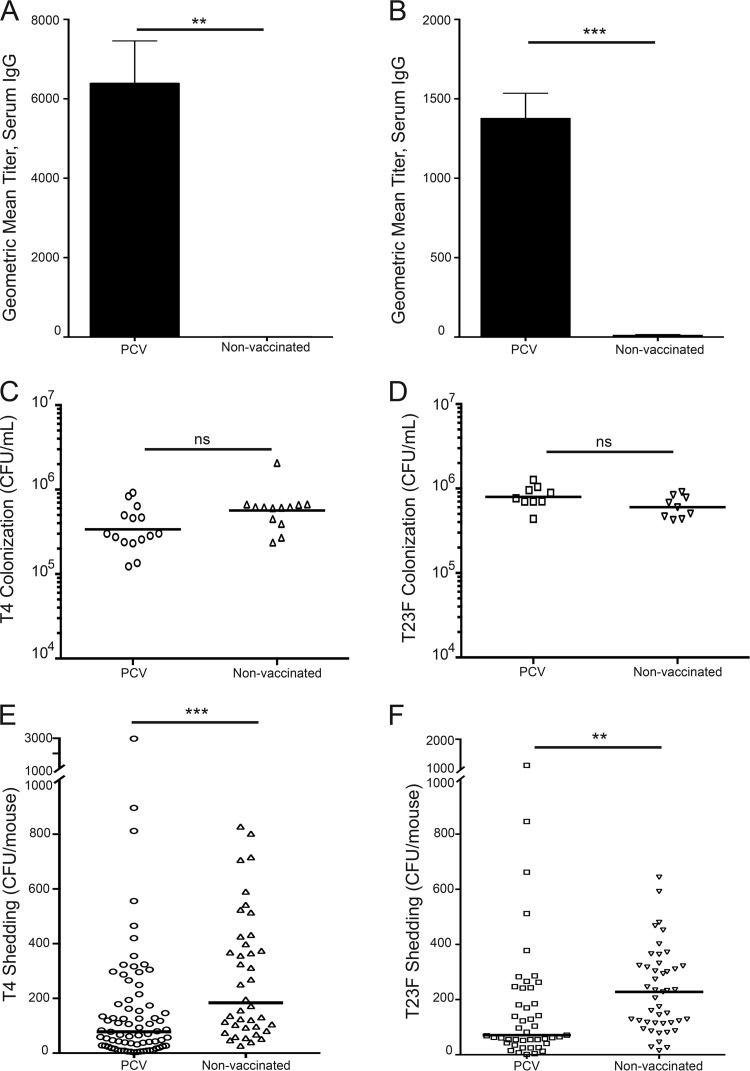
PCV13 vaccination induces an anti-pneumococcal polysaccharide (PPS) IgG serum response in dams, transferred to their pups, which reduces bacterial shedding but does not impact colonization. (A) Pups from PCV13-vaccinated mothers had higher anti-T4 PPS serum IgG titer, as assessed by PPS ELISA, than pups from non-vaccinated mothers. (B) Similarly, pups from PCV13-vaccinated mothers had higher anti-T23F PPS serum IgG titers than pups from non-vaccinated mothers. ELISA data are shown as mean titer + SEM. All pups were infected on day 4 of life, and serum was collected 5 days p.i. Pups were infected on day 4 of life, and colonization was assessed by URT lavage on day 5 p.i. (C) T4 and (D) T23F colonization of pups from vaccinated mothers did not differ from *S. pneumoniae* colonization of pups from non-vaccinated mothers. Pneumococcal shedding was collected on days 1 to 5 p.i. and is shown as median shedding, with each symbol representing the value from an individual pup on a single day. Pups from vaccinated mothers shed significantly less T4 (E) and T23F (F) than pups from non-vaccinated mothers. *n* ≥ 11 pups/group. **, *P* = 0.01; ***, *P* < 0.001 (Mann-Whitney test).

Maternal vaccination induced an anti-pneumococcal polysaccharide (PPS) antibody response in dams that is passed to their pups. Vaccinated pups had significantly higher serum IgG anti-T4 PPS (Fig. 2A) and anti-T23F PPS (Fig. 2B) titers compared to pups from non-vaccinated mothers, as evaluated by ELISA, using plates coated with purified capsular polysaccharide. This result was expected as both T4 and T23F PPS are included in PCV13. These anti-PPS titers were not sufficient to impact *S. pneumoniae* colonization in pups: T4 and T23F colonized vaccinated and non-vaccinated pups at similar levels (Fig. 2C and D). However, vaccinated pups shed significantly less T4 and T23F *S. pneumoniae* than pups from unvaccinated mothers (Fig. 2E and F). These data indicate that anti-PPS antibodies are sufficient to reduce *S. pneumoniae* shedding.

### Type-specific antibody is sufficient to reduce *S. pneumoniae* T23F shedding.

**FIG 3:**
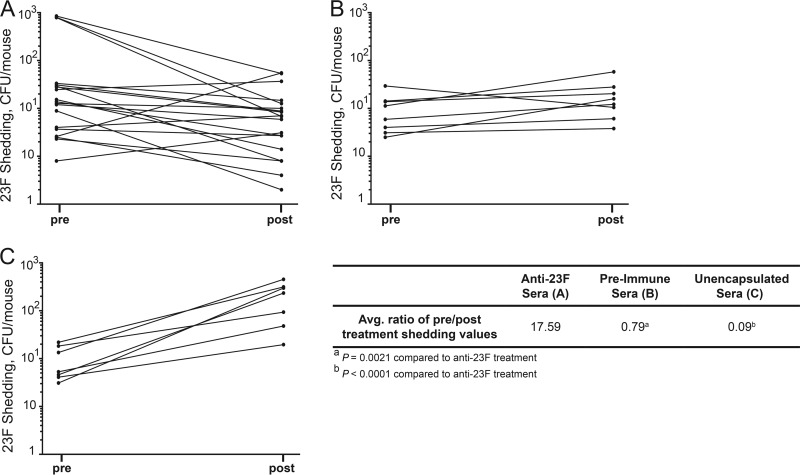
Type-specific antibody is sufficient to reduce *S. pneumoniae* 23F shedding. Pups were infected on day 4 of life with *S. pneumoniae* T23F and treated 24 to 48 h p.i. with rabbit sera (3 μl) by i.n. instillation. Shedding was assessed before and 6 h post-treatment. (A) Treatment with anti-T23F rabbit sera decreased shedding values, while treatment of littermates with pre-immune rabbit sera (B) or sera of a similar titer raised to the unencapsulated derivative strain (C) did not reduce shedding. Mean pre-treatment/post-treatment shedding ratios are significantly decreased in pups that received anti-T23F sera compared to pups that received pre-immune sera or sera raised to an isogenic unencapsulated strain. Each line represents one pup. Mean shedding ratios were compared by Mann-Whitney test.

To test whether anti-*S. pneumoniae* antibody can impact shedding, we infected (non-immune) pups on day 4 of life with *S. pneumoniae* T23F and then 24 or 48 h p.i. treated the pups by i.n. instillation with sera from a T23F-immunized rabbit (15). These experiments were done only with the T23F strain due to the availability of hyperimmune sera to the T23F strain. The pneumococcal nasal shedding sample was collected just prior to and 6 h after treatment with sera (Fig. 3), a time point chosen due to the short half-life of antibody delivered i.n. The average ratio of pre- to post-treatment shedding values was compared to T23F shedding from pups that received pre-immune rabbit sera. Importantly, in pilot studies we established that immune sera did not affect CFU quantification or URT colonization. Treatment with anti-T23F sera significantly reduced *S. pneumoniae* shedding (Fig. 3A) compared to shedding from littermate controls that received pre-immune control sera (Fig. 3B), which supports the hypothesis that anti-pneumococcal antibody is sufficient to decrease *S. pneumoniae* shedding. Interestingly, this effect of anti-T23F sera was most noticeable in pups with high shedding values pre-treatment (Fig. 3A). We further explored the role of type-specific antibody by comparing shedding from anti-T23F serum-treated pups to shedding from littermate controls that were treated with serum raised to an unencapsulated derivative of the T23F strain (Fig. 3C). Treatment with anti-T23F sera significantly decreased the shedding ratio compared to treatment with the sera raised against the unencapsulated strain (Fig. 3), highlighting the role of type-specific anti-PPS antibody in reduced shedding.

### Anti-PPS IgG is sufficient to reduce shedding and requires the agglutinating function of immunoglobulin.

**FIG 4:**
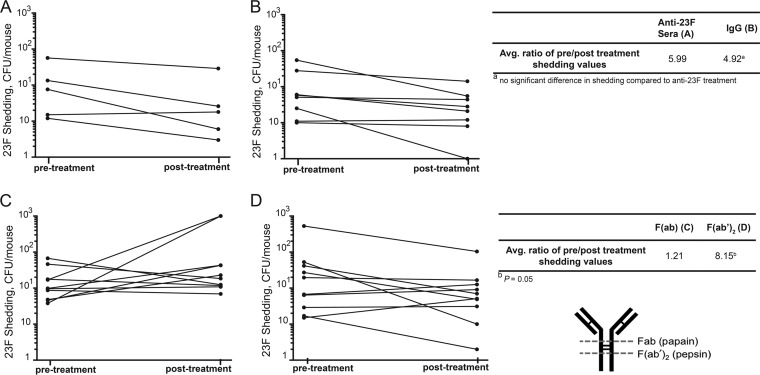
Anti-PPS IgG is sufficient to reduce pneumococcal shedding, and the agglutinating function of IgG is necessary to impact shedding. Pups were infected on day 4 of life with *S. pneumoniae* T23F and treated by i.n. instillation 24 to 48 h p.i. with anti-T23F rabbit sera or IgG purified from anti-23F rabbit sera. Shedding was assessed before and 6 h post-treatment. (A) Treatment with anti-23F rabbit sera decreased shedding, as did treatment of littermates with IgG from anti-T23F rabbit sera (B), and these pre-treatment/post-treatment shedding ratios are not different. However, treatment with Fab fragments did not impact shedding (C), whereas treatment of littermates with an equivalent quantity of F(ab′)_2_ (D) was sufficient to decrease shedding. *S. pneumoniae* T23F pre-treatment/post-treatment shedding ratios are significantly decreased in pups that received F(ab′)_2_ fragments generated from anti-T23F IgG compared to pups that received Fab fragments of anti-23F IgG. Each line represents one pup. Mean shedding ratios were compared by Mann-Whitney test.

To test if IgG was responsible for mediating the effect on shedding, we carried out similar experiments to those described above, where pups were infected on day 4 of life with *S. pneumoniae* T23F; 24 or 48 h p.i. they were treated with IgG purified from the anti-T23F rabbit serum. The IgG was sufficient to reduce shedding (Fig. 4A), similar to whole anti-T23F sera (Fig. 4B). The average ratios of pre- to post-treatment shedding values were not different between the two treatment groups (Fig. 4).

We next sought to define the mechanism by which anti-*S. pneumoniae* IgG decreases shedding. We recently reported that anti-*S. pneumoniae* IgG can block the establishment of pneumococcal colonization when adult mice were passively immunized prior to infection; this effect is independent of Fc-mediated opsonization and requires the agglutinating function of the antibody (16). To address whether agglutination is the mechanism by which IgG impacts shedding, purified anti-*S. pneumoniae* IgG was digested with pepsin to generate Fab fragments, which no longer have the agglutinating capacity of antibodies and lack the Fc region (16). As a control, the purified anti-*S. pneumoniae* IgG was also digested with papain to produce F(ab′)_2_ fragments in which the Fab fragments are still joined by a disulfide bond and are thus able to agglutinate but also lack the Fc region (Fig. 4). We previously confirmed that the F(ab′)_2_ fragments could agglutinate *S. pneumoniae in vitro*, but Fab fragments did not (16). *S. pneumoniae* T23F-infected pups were treated with equivalent amounts of Fab and F(ab′)_2_ fragments, and we compared the change in shedding pre- to post-treatment. Shedding either did not change or increased upon Fab treatment (Fig. 4C) while treatment of littermate controls with an equivalent quantity of F(ab′)_2_ resulted in decreased shedding values (Fig. 4D). The ratio of pre- to post-treatment shedding was significantly lower in F(ab′)_2_-treated pups compared to Fab-treated pups (Fig. 4). Collectively, these data indicate that anti-pneumococcal antibodies, and specifically anti-PPS antibody, is sufficient to decrease *S. pneumoniae* shedding from infected pups; furthermore, the data suggest that it is the agglutinating function of the IgG to type-specific PPS that is necessary to reduce shedding.

### Anti-*S. pneumoniae* antibodies are passed from mother to pup both *in utero* (systemic immunity) and post-natally (mucosal immunity).

To determine how and when the pups acquired anti-*S. pneumoniae* antibodies from their mothers, we set up a cross-foster experiment in which pups from an immune or vaccinated mother were placed with a non-immune mother and pups from a non-immune mother were placed with an immune or vaccinated mother within hours after birth. Pups remained with the foster mother for the duration of the experiment. On day 4 of life, the pups were infected with T4 or T23F, and shedding was assessed daily for 5 days p.i. Shedding values were significantly decreased from both immune and vaccinated pups with a non-immune mother and from non-immune pups with an immune or vaccinated mother compared to controls lacking anti-*S. pneumoniae* antibodies (Table 1), indicating that pups receive anti-*S. pneumoniae* antibodies from their mother both *in utero* and post-natally and at levels sufficient to impact pneumococcal shedding.

**TABLE 1:** Anti-pneumococcal immunity is passed from mother to pup both *in utero* and post-natally

Isolate	Immunity^a^		Median shedding, CFU/mouse (*P* value^b^)
	Dam	Pups (*n* ≥ 8)	
T23F	Non-immune	Non-immune	166
	Immune	Immune	65 (<0.0001)
	Non-immune	Immune	84 (0.0014)
	Immune	Non-immune	77 (0.0085)
T4	Non-immune	Non-immune	137
	Immune	Immune	54 (0.0054)
	Non-immune	Immune	35 (0.0001)
	Immune	Non-immune	64 (0.04)
T4	Non-vaccinated	Non-vaccinated	184
	Vaccinated	Vaccinated	78 (0.0009)
	Non-vaccinated	Vaccinated	39 (<0.0001)
	Vaccinated	Non-vaccinated	48 (0.0002)

a “Immune” refers to mothers immunized by colonization and “Vaccinated” refers to mothers immunized by PCV administration.

b Compared to median shedding of non-immune/non-vaccinated pups with a non-immune/non-vaccinated mother (Mann-Whitney test).

### Anti-pneumococcal immunity and anti-PPS vaccination significantly reduce transmission of *S. pneumoniae* T4.

Finally, we addressed the role of anti-pneumococcal immunity on close-contact transmission of *S. pneumoniae* from one infected pup to another susceptible littermate. One-half of a litter was infected with T4 (“index” mice), and the other half was not infected (“contact” mice). After a 10-day exposure period, all pups were euthanized and assessed for colonization. Each colonized contact pup was considered a transmission event. Only transmission of *S. pneumoniae* T4 was assessed, as T4 transmission rates, but not T23F rates, were high enough to allow for meaningful comparsion. Immunity to T4 significantly decreased transmission within the litters (Table 2), and transmission was also significantly reduced among pups from PCV13-vaccinated mothers (Table 2), as no transmission events occurred in either immune or vaccinated litters. These data indicate that anti-PPS immunity is sufficient to decrease intralitter transmission. To address whether it is the anti-pneumococcal immunity of the index pup (exit stage) or the contact pup (entry state) that is blocking transmission, we set up mixed-litter transmission studies wherein only the index or contact mice were T4 immune (and all dams were non-immune). There were no *S. pneumoniae* transmission events in either scenario (Table 2), indicating that both decreased pneumococcal shedding and prevention of *S. pneumoniae* acquisition are key factors in reducing the transmission of *S. pneumoniae*.

**TABLE 2:** Anti-pneumococcal immunity significantly reduces transmission

Index condition^a^	Contact condition^a^	No. of contacts		% of infected contact mice (*P* value^b^)
		Infected	Noninfected	
Non-immune	Non-immune	9	22	29^c^
Immune	Immune	0	16	0 (0.019)
Vaccinated	Vaccinated	0	14	0 (0.039)
Immune	Non-immune	0	13	0 (0.041)
Non-immune	Immune	0	12	0 (0.044)

a “Immune” refers to mothers immunized by colonization and “Vaccinated” refers to mothers immunized by PCV administration.

b Compared to non-immune contact mice with non-immune index mice condition (Fisher's exact test).

c See reference 12.

## DISCUSSION

The aim of this study was to determine how immunity to *S. pneumoniae* impacts host-to-host transmission. We focused on this aspect of the infectious process because extensive worldwide experience with PCV has demonstrated that its main contribution to lowering the burden of pneumococcal disease is by reduced transmission (for *S. pneumoniae* serotypes included in the vaccine formulation [9, 10]). Any novel and potentially more broadly acting pneumococcal vaccine would likewise need to impact transmission to be as effective as the PCV.

Our findings in an infant mouse model showed that anti-*S. pneumoniae* immunity decreases pneumococcal shedding from infected pups. Since URT colonization densities were similar among all pups in this study, we eliminated the lower density of bacteria in the URT as a cause of decreased nasal shedding of *S. pneumoniae* in immune mice. Shedding is variable from pup to pup and time point to time point, as previously documented (12); our comparisons are therefore based on the analysis of many pups over multiple days. The decrease in mean bacterial shedding was correlated with a complete loss of within-litter *S. pneumoniae* transmission and was consistent with prior observations on the association of high shedding values and intralitter transmission (11–13). Here we report decreased *S. pneumoniae* shedding and transmission in pups from mothers that were immune due to either prior colonization or PCV vaccination, making it unlikely that indirect effects (such as a maternally acquired change in the URT flora of pups from previously colonized mothers)—rather than immunity—account for our findings.

Passive immunization with type-specific antisera, IgG, and IgG fragments showed that antibody was sufficient to decrease pneumococcal shedding and that this effect could be attributed to the agglutinating function of IgG. In a study in which the nasal spaces of recently colonized mice were viewed in tissue sections, pneumococci are seen in much larger clusters in immunized compared to nonimmunized animals (16). Agglutination may facilitate more rapid clearance of bacteria that detach from mucosal surfaces of the upper airways and thereby decrease bacterial counts in a shedding assay. These bacterial aggregates might be more easily removed by mucociliary flow rather than expelled from nares into the environment where they can access a new host. Anti-*S. pneumoniae* IgG is able to block the acquisition of colonization in a dose-dependent manner (if present in sufficient amounts at the time of challenge), and this effect is dependent upon its agglutinating function (16). Observations in the present report suggest that in addition to its ability to block acquisition, the agglutinating effect of mucosal anti-*S. pneumoniae* IgG decreases the number of shed bacteria below a threshold critical for transmission.

The anti-*S. pneumoniae* antibody measured in the pups was primarily IgG, and therefore, our data support the role of IgG in decreasing shedding and blocking transmission—an observation consistent with the immune response to PCV, which is administered parenterally and stimulates a robust IgG response (17). Accordingly, a human challenge study showed that anti-capsular antibody generated after vaccination with PCV is sufficient to protect adults against challenge with an *S. pneumoniae* strain of a serotype in the vaccine (15). Of note, in that study, anti-PPS IgG induced by parenteral vaccination with PCV was measureable in nasal wash samples from human volunteers (15), which correlates with our findings of anti-*S. pneumoniae* IgG detected in URT lavage samples from immune mice. In the experimental human pneumococcal challenge study, protection from acquisition correlated with the agglutinating activity of nasal wash samples (15). Anti-pneumococcal mucosal IgG is also present following URT *S. pneumoniae* carriage (18), and our present report demonstrated that anti-*S. pneumoniae* IgG is induced by colonization in adult mice, mucosal IgG was passed from dam to pup, and IgG was capable of decreasing pneumococcal shedding and blocking transmission.

In this study, the presence of maternally acquired anti-*S. pneumoniae* antibody did not affect the colonization density of *S. pneumoniae* in the URT of infant mice, nor did it prevent the acquisition of colonization following i.n. inoculation with ~10^3^ organisms. Humoral immunity has minimal impact on the numbers of mucosal pneumococci once colonization is established (19, 20). We have previously reported that passive i.n. immunization with type-specific antibody could inhibit acquisition and transmission in adult mice (16); infant mice, however, are more susceptible to colonization with *S. pneumoniae* and can be colonized by a dose of fewer than 10 pneumococci (13)—an amount that is approximately 100-fold fewer than what is required to establish similar levels of colonization in adults. It appears that in infant mice, the amount of mucosal IgG was insufficient to prevent pneumococcal acquisition by i.n. inoculation with the inoculum size tested. In contrast, evidence suggests that the levels of antibody present were adequate to block natural acquisition within the litter, as there was no transmission from non-immune index to immune contact pups. One explanation for the difference in acquisition by inoculation but not transmission might be a matter of the challenge dose: our recent study on the population bottleneck in transmission shows that colonization is most often established only by a single pneumococcal cell/chain (13).

The primary goal of this study was not to test protection via maternal immunity or immunization—a topic that has been the focus of much clinical investigation (reviewed in reference 21). Pneumococcal carriage is common by 2 months of age in the developing world and, in some locations, this pathogen is second only to group B *Streptococcus* as a cause of invasive bacterial infection in infants (22). Although vaccination during pregnancy leads to high anti-PPS levels in infants, the efficacy of maternal immunization for the prevention of carriage or disease early in childhood is still debated and under investigation. Studies in mouse models demonstrate the protection of newborns from pneumococcal disease through maternal immunization (23) and support a role for the transfer of protective antibody from breast milk or from the serum pool *in utero* (24). The cross-foster experiments in our study, in which immune pups were raised by a non-immune dam or non-immune pups by an immune dam, both showed decreased shedding compared to non-immune pups raised by a non-immune dam, confirming that protective antibody can be transferred both *in utero* and post-natally, in accordance with published observations (25, 26).

A better understanding of factors that impact *S. pneumoniae* transmission, including those that affect bacterial colonization, shedding, and acquisition, has implications for future vaccine development. In this study, we provide a mechanistic explanation for the “herd immunity” effect, seen widely after the introduction of PCV. By highlighting the importance of reduced shedding and bacterial acquisition in the *S. pneumoniae* transmission cycle, we have identified vulnerable points for intervention. This study provides a more comprehensive analysis of how active immunity to *S. pneumoniae* and, in particular, antibody to PPS, impact transmission and promote herd immunity. PCV-induced type-specific immunity was shown here to be sufficient to reduce shedding and prevent transmission for isolates of two serotypes included in the vaccine formulation. Moreover, in passive immunization assays, high-titer serum raised against whole pneumococci, but not its unencapsulated derivative, decreased pneumococcal shedding. These results point to a requirement for type-specific immunity in blocking pneumococcal transmission. We cannot, however, exclude the possibility that natural infection presents a distinct set of antigens not targeted by the immune serum tested herein. PPS is an immunodominant and abundant antigen, and physiological levels of type-specific antibody readily agglutinate encapsulated pneumococci (15). It remains unclear, therefore, whether there are other serotype-independent pneumococcal targets of agglutinating antibody that could similarly reduce shedding, block acquisition, and prevent transmission.

Finally, there are few tractable models available for examining host factors in transmission, especially involving infections of the respiratory tract. The mechanistic insight from our investigation of *S. pneumoniae* may be relevant to the role of immunity in the transmission of other infectious agents.

## MATERIALS AND METHODS

### Bacterial strains and culture.

*Streptococcus pneumoniae* isolate P2406 (streptomycin resistant [Str^r^]) is a capsule type 4 (T4) derivative of TIGR4 (12). Strain P1397 is an Str^r^ derivative of P1121, a capsule type 23F (T23F) strain isolated from the nasopharynx in a human experimental carriage study (27). *S. pneumoniae* strains were grown in tryptic soy (TS) broth (BD, Franklin Lakes, NJ) at 37°C without aeration to an optical density at 620 nm (OD_620_) of 1.0 or incubated on TS agar plates supplemented with 100 μl of catalase (30,000 U/ml; Worthington Biochemical) and Str (200 μg/ml) at 37°C in 5% CO_2_, overnight.

### Mouse studies.

Wild-type C57BL/6J mice (Jackson Laboratories, Bar Harbor, ME) were used in all experiments. Mice were maintained and bred in a conventional animal facility. Pups were housed with a dam for the course of the studies. Following infection, all mice appeared healthy, showed normal activity, and gained weight similar to uninfected controls. These studies were conducted in accordance with the recommendations of the *Guide for the Care and Use of Laboratory Animals*. All mouse studies were approved by the Institutional Animal Care and Use Committee of the New York University Medical Center. All procedures were in compliance with *Biosafety in Microbiological and Biomedical Laboratories*.

### Maternal immunization.

Anti-pneumococcal immunity was induced in adult female mice via colonization. Briefly, mice were infected intranasally (i.n.) with 10^7^ CFU of either a T23F or T4 strain in 10 μl of sterile phosphate-buffered saline (PBS) by instillation on the nares with a blunt pipette tip, without anesthesia. The female mice were then paired with males, and their litters were used in experiments to assess the role of immunity. The presence of anti-pneumococcal IgG in serum of mothers and their pups was confirmed by an ELISA using whole bacteria (as described below).

### Maternal vaccination.

Adult, female mice were vaccinated with PCV13 (Prevnar13; Pfizer) by intramuscular injection (50 μl), two times at an interval of 21 days (28). Control mice received PBS. The mice were then bred, and pups were utilized in experiments. The presence of anti-pneumococcal capsule IgG in serum of mothers and their pups was confirmed by polysaccharide ELISA.

### Shedding and colonization.

Four-day-old pups were infected with 10^3^ CFU of *S. pneumoniae* in 3 μl of PBS by i.n. instillation with a blunt pipette tip, without anesthesia, and returned to their mother for the duration of the experiment. To assess daily nasal shedding of *S. pneumoniae*, the nose of the pup was gently tapped 20 times evenly across a TS-Str plate; the sample was spread with a sterile cotton swab, and the colonies were enumerated after overnight incubation. Due to variation in day-to-day shedding, values collected daily over the 5-day infection period were combined for statistical analysis to allow for meaningful comparisons. At the end of the study, pups were euthanized by CO_2_ asphyxiation and cardiac puncture. Upper respiratory tract (URT) colonization of *S. pneumoniae* was determined as described previously (11). Briefly, the trachea was lavaged with 200 μl of sterile PBS, which was collected from the nose. Tenfold serial dilutions of the lavage sample were plated to TS-Str and enumerated after overnight incubation.

### Intranasal treatment with serum and IgG.

Pups were infected with T23F strain by i.n. instillation, as described above. At 24 to 48 h p.i., the litters were divided, half of the pups were treated i.n. with 3 μl of serum from rabbits hyperimmunized to heat-killed, whole-cell *S. pneumoniae* strain P1121 (type 23F), and the other half received 3 μl of serum from rabbits immunized to a capsule-deficient derivative strain (serum generation described in reference 15) or 3 μl of pre-immune sera. IgG titers of the P1121 antiserum and sera raised to its unencapsulated derivative were not significantly different (15). Pneumococcal shedding was assessed before and 6 h after antiserum treatment. In other experiments, pups were treated i.n. with 10 μg of IgG purified from the anti-P1121 serum or with 10 μg Fab or F(ab′)_2_ IgG fragments suspended in 3 μl of PBS. IgG was purified from the rabbit serum as previously described (16) following the manufacturer’s protocol with the following modifications: serum was diluted 4:1 in binding buffer and applied to a HiTrap protein G column (GE Healthcare Bio-Sciences, Pittsburgh, PA), and IgG eluent was buffer exchanged into PBS using Amicon 10,000 molecular weight cutoff (MWCO) centrifugal filters (EMD Millipore, Billerica, MA). IgG was digested into Fab and F(ab′)_2_ fragments as described previously (16). IgG and Fab samples were run on Bolt 4 to 12% Bis-Tris Plus gels (Invitrogen, Carlsbad, CA) and stained with SimplyBlue SafeStain (Invitrogen, Carlsbad, CA) to confirm IgG purification and complete digestion. *In vitro* agglutination of *S. pneumoniae* by F(ab′)_2_, but not Fab, fragments was confirmed as described previously (16).

### Intralitter transmission.

Pneumococcal transmission among pups was carried out as previously described (12). Four-day-old pups were infected with 10^3^ CFU of *S. pneumoniae* in 3 μl of PBS by i.n. instillation and returned to the litter for the duration of the experiment. The ratio of infected (“index”) to uninfected (“contact”) pups was 1:1 in all experiments. On day 14 of life, all pups were euthanized and a URT lavage was carried out to quantify *S. pneumoniae* colonization of all pups. The transmission rate was determined by the proportion of contact mice that had become infected during the course of the experiment.

### ELISA.

An ELISA to assess the presence of anti-pneumococcal IgG in the serum of immunized mothers and their pups, using whole bacteria as the capture antigen, was done as previously described (29). Briefly, Immulon 2HB flat-bottom microtiter plates were coated with 100 μl of PBS-washed whole bacteria (*S. pneumoniae* T23F or T4), diluted in coating buffer (0.015 M Na_2_CO_3_, 0.035 M NaHCO_3_) to a final OD_620_ of 0.1, and incubated at 4°C overnight. The plates were then washed three times with wash solution (0.05% Brij-35 Surfact-Amps [Thermo, Fisher Scientific]–PBS) and blocked for 1 h at room temperature with 1% bovine serum albumin (BSA)–PBS. Serial serum dilutions, in PBS, were applied to the plates and incubated at 4°C overnight. The plates were then washed three times with wash solution. Mouse IgG was detected with alkaline phosphatase (AP)-conjugated goat anti-mouse IgG antibody (Sigma A5153) at a 1:4,000 dilution in PBS, incubated at 37°C for 1.5 h. The plates were then washed three times with wash buffer. The plate was developed with *p*NPP substrate tablets (Kirkegaard & Perry Laboratories Inc., Gaithersburg, MD) dissolved in diethanolamine (DEA [Thermo, Fisher Scientific]). Absorbance at 415 nm was read at 1 h. The endpoint titers were determined by calculating the dilution at which the absorbance is equal to an OD_415_ of 0.1.

Pneumococcal polysaccharide (PPS) ELISA assessed the presence of anti-pneumococcal capsule antibodies in the serum of mothers and their pups vaccinated with PCV. Purified T23F or T4 pneumococcal polysaccharide (ATCC) was diluted to a final concentration of 5 μg/ml in PBS and applied to Immulon 1B flat-bottom microtiter plates. Plates were incubated overnight at 4°C and then washed three times with PBS and blocked for 1 h at 37°C with 1% BSA–PBS. Serially diluted serum samples, in 1% BSA–PBS, were applied to the plate and incubated at 37°C for 2 h. The plate was washed with PBS, and 100 μl of AP-conjugated goat anti-mouse IgG (diluted 1:4,000 in 1% BSA–PBS) was applied to each well. The plate was incubated at 37°C for 1.5 h. The wells were washed with PBS, and the plate was developed as described above.

### Statistical analysis.

Statistical analysis was done using GraphPad Prism 6.0 (GraphPad Software, Inc., San Diego, CA). Unless indicated otherwise, differences between two groups were compared using Mann-Whitney test, where *P* ≤ 0.05 was considered significant.
